# *Populus alba* L., an Autochthonous Species of Spain: A Source for Cellulose Nanofibers by Chemical Pretreatment

**DOI:** 10.3390/polym14010068

**Published:** 2021-12-25

**Authors:** David Ibarra, Raquel Martín-Sampedro, Bernd Wicklein, Antonio M. Borrero-López, Concepción Valencia, Ana Valdehíta, José M. Navas, María E. Eugenio

**Affiliations:** 1 Forest Research Center (INIA, CSIC), Ctra. de la Coruña Km 7.5, 28040 Madrid, Spain; ibarra.david@inia.es (D.I.); raquel.martin@inia.es (R.M.-S.); 2 Materials Science Institute of Madrid (ICMM), Consejo Superior de Investigaciones Científicas (CSIC), Sor Juana Inés de la Cruz 3, 28049 Madrid, Spain; bernd@icmm.csic.es; 3 Pro2TecS—Chemical Process and Product Technology Research Centre, Departamento de Ingeniería Química, ETSI, Campus de “El Carmen”, Universidad de Huelva, 21071 Huelva, Spain; am.borrero@diq.uhu.es (A.M.B.-L.); barragan@diq.uhu.es (C.V.); 4 Environment and Agronomy Department (INIA, CSIC), Ctra. de la Coruña Km 7.5, 28040 Madrid, Spain; ana.valdehita@inia.es (A.V.); jmnavas@inia.es (J.M.N.)

**Keywords:** cellulose nanofibers, chemical pretreatment, cytotoxicity, nanocellulose, *Populus alba* L., rheology

## Abstract

In order to identify new sustainable sources for producing cellulose nanofibers (CNFs), fast-growing poplar (*Populus alba* L.) wood was evaluated herein. For that purpose, bleached poplar kraft pulp was produced and submitted to TEMPO (2,2,6,6-tetramethylpiperidine-1-oxyl radical) mediated oxidation (TEMPO-ox) chemical pretreatment followed by microfluidization. The resulting CNFs were thoroughly characterized, including a rheological study at different pH values. Poplar CNFs showed properties comparable to eucalypt CNFs (reference material for CNFs production), showing high carboxylate content (1048 ± 128 µmol g^−1^), fibrillation yield (87.3% ± 8.1%), optical transmittance (83% at 700 nm) and thermal stability (up to more than 200 °C). Regarding the rheological study, whereas pH from 4 to 10 did not produce significant changes in rheological behavior, a reduction of pH down to 1 led to an order-of-magnitude increase on the viscoelastic functions. Therefore, poplar CNF shows potential in the pH-sensitive hydrogels application field. Finally, the possible ecotoxicity of poplar CNF was assessed. The decrease in cell viability was very low so that only concentrations causing a 10% cytotoxicity could be calculated for the assay detecting alterations in cell metabolism (10 µg mL^−1^) and plasma membrane integrity (60 µg mL^−1^).

## 1. Introduction

The search for new and abundant sources of lignocellulosic biomass to supply biorefineries, for bioproducts and biofuels production, is currently on the rise in order to eliminate our dependence on fossil fuels [1]. In this context, the Short Rotation Coppice system (SRC) is one of the most effective options for producing lignocellulosic biomass. One of the advantages of the SRC system, from a logistic point of view, is its tendency to be localized, in space and time [2]. Among the raw materials suitable for obtaining biomass through SRC are species within the genus *Populus* [3]. Moreover, these species show a hardness and plasticity suitable for growing in marginal areas affected by salinity, high temperatures and dry climates, such as Mediterranean conditions [3]. These materials have contents of 42–49% in cellulose, higher than in other hardwood such as eucalypt [4], together with 16–23% in hemicellulose and 21–29% in lignin [5,6]. The high sugar content of different species and hybrids of genus *Populus* has promoted their use as a source of biomass in lignocellulosic biorefineries. For example, these raw materials have been used to obtain fermentable sugars [5,6], from which bioethanol [7] and xylitol are produced [8]. Moreover, different bioproducts such as activated carbon for water defluorination [9] or adhesives [10] have also been obtained from its ligninolytic fraction. However, these materials have scarcely been studied to produce cellulose-based products, such as nanocellulose.

Nanocellulose is currently in demand due not only to its specific properties, i.e., renewably sourced, biodegradable, and biocompatible, but also to its excellent characteristics [11]. In particular, nanocellulose and more specifically cellulose nanofibers (CNFs) are characterized by properties such as transparency, low density, high mechanical resistance, thermal and surface stability, and versatility of chemical modification [12]. These characteristics make it an ideal material to be used in several applications such as paper; construction materials; packaging; paints; medical applications; and flexible, printed electronics among others [13,14,15,16].

CNFs form long, flexible fiber networks, which are composed of microfibrils with a nano-size diameter that depends on the method used to obtain them [17]. CNFs can be obtained by mechanical microfibrillation processes (using refiner, grinder, homogenizer, microfluidizer, etc.) from cellulose pulp, although with high energy consumption. Chemical, biological and mechanical pretreatments have been studied to reduce the energy consumption of the microfibrillation process in order to allow the implementation of the CNFs production at an industrial scale [14]. Among the chemical pretreatments, TEMPO (2,2,6,6-tetramethylpiperidine-1-oxyl radical) mediated oxidation (TEMPO-ox) has been selected as one of the most effective in reducing the energy consumption in the further mechanical microfibrillation treatment. Under alkaline conditions, the system TEMPO/NaBr/NaClO can oxidize the C6-primary hydroxyl groups found on the surface of cellulose microfibers to carboxyl groups [18]. To carry out this reaction, only NaClO and NaOH are consumed [19]. This reaction negatively charges the fibril surface creating a repulsive effect between adjacent fibrils. Consequently, the efficiency of the separation of the individual nanofibers is increased in the subsequent mechanical microfibrillation treatment, reducing the energy consumed in the whole process [20].

Not only the technology used but also the starting raw material has a marked influence on the characteristic of the CNFs obtained [21]. Therefore, the search for new raw materials as alternatives to traditional woody species (e.g., eucalypt, birch, pine, spruce, etc.) used for CNFs production is a research line of interest to open even more the range of applications of the CNF [14]. As it was mentioned before, to our knowledge, only a few studies about CNFs production from different species and hybrids of genus *Populus* have been reported and none of them have used poplar biomass obtained by SRC or TEMPO-ox process as pre-treatment. For example, Qi et al. [22] have used poplar residues (branches) taken from the suburbs of the city of TaiAn (China). Then, nanocellulose was produced from chemically purified cellulose via combined mechanical treatments of grinding and high-pressure homogenization. The nanocellulose obtained by these authors was applied in the preparation of nanopaper. Zhao et al. [23] studied the thermostability of nanocellulose obtained from poplar wood supplied by a pilot plant located in Jiangsu, China. These authors produced nanocellulose from purified cellulose of poplar wood via mechanical treatment with high pressure-grinding. Wu et al. [24] have studied the nanocellulose production from poplar (*Populus tomentosa*) catkin fiber via sonication. Finally, Ibarra et al. [25] produced microfibrillated cellulose from fast-growing poplar using a physical pretreatment (PFI refining) before the microfibrillation stage, showing similar properties to that obtained from a commercial industrial eucalypt pulp. Then, films from this microfibrillated cellulose were produced with high mechanical properties and low wettability.

Considering all the above, the objective of this work is to prove the use of *Populus alba* L. “PO-10-10-20”, an autochthonous genotype of Spain currently tested in SRC plantations for biomass production, as a source for CNFs production. To this end, TEMPO-ox reaction as pre-treatment and subsequent defibrillation in a high-pressure microfluidizer was used. Resulting CNFs were thoroughly characterized (carboxylate content, fibrillation yield, optical transmittance and thermal stability), including X-ray diffraction and Fourier transform infrared spectroscopy (FTIR). Moreover, an extensive rheological study modifying the pH of the CNFs was also carried out. Finally, the possible ecotoxicity of poplar CNFs was also assessed.

## 2. Materials and Methods

### 2.1. Raw Materials and Chemicals

The autochthonous genotype of *P. alba* L., “PO-10-10-20”, was supplied by the Silviculture and Forest Management Department of INIA, CSIC (Madrid, Spain). The short rotation planting protocol in Mediterranean conditions described in Sixto et al. [26] was used for planting this genotype. The plantation was cut after three years of growth (first rotation) and the resulting material was used to produce the CNF.

CNF from eucalypt was produced in previous work [21] and was used as a reference hydrogel.

Sodium hypochlorite solution (NaClO), TEMPO, sodium bromide (NaBr), hydrochloric acid (HCl), sodium hidroxyde (NaOH), sodium sulfide (Na_2_S), were purchased from Sigma–Aldrich (Steinheim, Germany) and magnesium sulphate (MgSO_4_), hydrogen peroxide (H_2_O_2_) and diethylenetriaminepentaacetic acid (DTPA) from Merck Life Science S.L (Madrid, Spain).

For cell culture, in the cytoxicity analysis, L-Glutamine (200 mM), penicillin and streptomycin (P/S) (10,000 U mL^−1^ each), trypsin-ethylene diamine tetraacetic acid (EDTA) (200 mg/mL), and Eagle’s Minimum Essential Medium (EMEM) cell culture and non-essential aminoacids, (NEAA) were purchased from Lonza (Barcelona, Spain). Phenol-red free serum-free Minimum Essential Medium (MEM) was supplied by Gibco (Life Technologies, Madrid, Spain). Among the reactants used for determining cytotoxicity, AlamarBlue reagent and 5-carboxyfluorescein diacetate, acetoxy methyl ester (CFDA-AM) and neutral red were purchased from Invitrogen (Madrid, Spain).

### 2.2. Bleached Pulp Production

*P. alba* L. “PO-10-10-20” chips were subjected to a kraft pulping process using a 15-litre batch cylindrical reactor which incorporates a computer-controlled electric jacket heater. To ensure proper agitation, the reactor was equipped with a rotating shaft. Pulping conditions were as follows [27]: 17% active alkali, 20% sulphate (related to sulphur content), 170 °C, 120 min, and a liquid/solid ratio of 7:1. The resulting material from the pulping was subjected to a wet disintegration process for 30,000 revolutions and then screened through a 0.16 mm mesh.

After pulping, a standard industrial totally chlorine-free (TCF) bleaching sequence consisting of OOQPoP was carried out, where O was a delignification stage with oxygen (held twice), Q was a chelating stage, and Po and P were hydrogen peroxide bleaching stages with or without oxygen, respectively. Conditions of both oxygen delignification stages were: 6 MPa of oxygen pressure, 60 min, 98 °C, 0.5% MgSO_4_ o.d.p. (oven-dry pulp), 1.5% NaOH o.d.p., and 20% of consistency. The oxygen steps were carried out using the same reactor described above for the pulping process. The chelating step was performed using 0.3% DTPA at 85 °C for 60 min (pH 5–6). Finally, bleaching conditions in the first hydrogen peroxide step was: 2% NaOH, 0.5% Na_2_Si_2_O_3_, 0.1% MgSO_4_ and 3% H_2_O_2_ for 140 min at 105 °C pressurized with oxygen at 6 kg cm^−2^ while the second step was carried out using the same chemical reagents but during 180 min at 98 °C without pressure [28]. Pulps samples were washed after each stage using distilled water and stored at 4 °C.

### 2.3. CNF Production

The bleached pulp obtained from *P. alba* L. “PO-10-10-20”, was first subjected to a demineralization process. For this purpose, 45 g of bleached pulp was soaked in a diluted solution of HCl (4500 mL) at pH ∼2 for 20 min at room temperature with mechanical stirring. Subsequently, the pulp was washed with distilled water repeatedly by filtration. For CNF production, the already demineralized pulp was subjected to an oxidation process with TEMPO as described by [18]. Thus, the pulp was dispersed in a solution containing 0.016 g/g o.d.p. of TEMPO and 0.1 g/g o.d.p. of NaBr to which a solution of 10 mmol/g o.d.p. NaClO was added maintaining the pH of the total dispersion at 10 by adding 0.5 M NaOH, until no decrease in pH was observed. After that, the dispersion containing the oxidized fibers was filtered and washed up to pH 7, adjusted to a 2% consistency and stored at 4 °C. To obtain the final product (CNF), the resulting dispersion at 2% was subjected to a mechanical process, passing three times by a high-pressure microfluidizer (Microfluidizer M-110EH, Microfluidics Corp., Westwood, MA, USA) using interaction chambers of 100 and 200 µm. Obtained CNF (referenced as poplar CNF) was stored at 4 °C.

### 2.4. Pulp and CNF Characterization

#### 2.4.1. Chemical Composition

The procedure provided by The National Renewable Energies Laboratory [29] was used to determine the chemical composition of poplar pulp and CNF. Thus, the analysis of carbohydrates and lignin was carried out by means of acid hydrolysis. From the liquid fraction resulting from the hydrolysis, carbohydrates were quantified by liquid chromatography (HPLC) on an Agilent Technologies 1260 HPLC (Agilent, Waldbronn, Germany) using an Agilent Hi-PlexPb column operated at 70 °C with ultrapure water as a pumped mobile phase at a rate of 0.6 mL min^−1^. This HPLC was equipped with a refractive index detector (Agilent, Waldbronn, Germany). On the other hand, the residual solid from hydrolysis is denominated insoluble acid lignin (Klason lignin).

#### 2.4.2. Fibrillation Yield

The determination of the fibrillation yield of poplar CNF was carried out by centrifugation. For this purpose, a 0.1% CNF dispersion was centrifuged for 20 min at 4500 rpm in order to separate the nanofibrillated material (present in the supernatant fraction) from the partially or non-fibrillated one (sediment) as described by Besbes et al. [30]. The fibrillation yield (nanofibrillated part) was calculated by Equation (1):

Yield (%) = 100 (1 − (Wp/Ws)(1)

where Wp is the weight of the dry sediment after centrifugation (g o.d.p.) and Wp is the weight of the CNF sample before centrifugation (g o.d.p.). Mean values and standard deviations were calculated from triplicates.

#### 2.4.3. Optical Transmittance

To determine the optical transmittance of the sample, a 0.1% (*w*/*v*) dispersion of poplar CNF was prepared in water. The transmittance was measured using a Lambda 365 UV—vis spectrometer (PerkinElmer, Waltham, MA, USA) from 285 to 750 nm.

#### 2.4.4. Polymerization Degree

The polymerization degree (DPv) of the pulp and the poplar CNF, was calculated using the Mark–Houwink Equation (2):

μ = Kp × DPv^a^ (2)

where μ is the viscosity measured in the samples according to ISO 5351-2 using a cuprietilendiamine (CED) solution and where a and Kp are constant (0.80 mL g^−1^ and 1.70, respectively) [31]. Mean values and standard deviations were calculated from triplicates.

#### 2.4.5. Carboxylate Content

The conductometric titration method described by Fras et al. [32] with some modifications was used for the determination of the carboxylate content of the poplar CNF. By means of an ion-exchange treatment, the sodium carboxylate groups present in the sample were the first converted into free carboxyl. For this purpose, 0.10 g o.d.p. of the sample was dispersed in 185 mL of a 1 mM NaCl solution by stirring under a nitrogen atmosphere for 30 min at 25 °C. By adding 0.1 M HCl the pH of the solution was adjusted to <3. Subsequently, the titration was performed with the addition of 0.05 M NaOH at 30 s intervals. A Crison GLP 31 conductivity meter (Crison, L’Hospitalet de Llobregat, Spain) was used to measure conductivity. Equation (3) was used to determine the carboxylate content of the sample:

Carboxylate content (mmol L^−1^) = c (V2−V1)/w (3)

where c is the concentration of NaOH (M), V1 and V2 are the volume of NaOH (mL) consumed in the first and second point of intersection respectively, w is the dry weight of the sample (g o.d.p.). Mean values and standard deviations were calculated from triplicates.

#### 2.4.6. X-ray Diffraction (XRD)

The crystallinity of the poplar pulp and CNF was analyzed by X-ray powder diffraction. The equipment used was Bruker D8 Advance (Bruker, Billerica, MA, USA) which was equipped with a Cu anode (radiation CuKα) and a Ni filter. The preparation of the sample for such an analysis requires the preparation of a film by casting. Equation (4) was used to calculate the crystallinity index (CI) by the Segal method:

CI = [(I_002_−I_AM_)/I_002_]100 (4)

where I_AM_ is the intensity at approximately 2θ = 18.5° attributed to the amorphous phase and I_002_ is the intensity of the (002) reflection.

Using the reflection (002) according to the Scherrer equation, the crystallite size t was calculated:

t = k × λ/(β × cos θ)(5)

where λ is the radiation wavelength, k = 0.9 is the correction factor, θ is the diffraction angle and β is the full width at half maximum.

#### 2.4.7. Cytotoxicity Analysis

The stock suspension of CNFs (poplar CNF and eucalypt CNF used as reference) were prepared following the NANOGENOTOX dispersion [33]. CNFs cytotoxicity was studied in vitro by means of the topminnow fish hepatoma cell line (PLHC-1) obtained from the American Type Culture Collection (Manassas, VA, USA). PLHC-1 was maintained in EMEM (with NEAA and Na Pyruvate) supplemented with 1% L-glutamine, 1% P/S and 5% FBS at 30 °C with 5% CO_2_.

PLHC-1 cells were seeded in flat-bottomed 96-well plates (Greiner Bio-One GmbH, Germany) at a density of 5 × 10^4^ cells mL^−1^. Following 24 h, cells were exposed to a concentration range of 0 to 256 µg mL^−1^ of CNFs (dilution factor 2) for 72 h. Although exposure concentrations may not reflect environmentally realistic scenarios, the concentration range chosen allowed us to construct the logistic curve from which inhibitory concentration (IC, concentration causing a given percentage of cell growth inhibition) values were calculated. Control wells were treated with medium or medium plus BSA. Sodium dodecyl sulphate (SDS, 65.84 to 500 µM, dilution factor 1.5) served as a positive cytotoxicity control. At least three independent experiments were carried out with a minimum of three replicates of each CNFs concentration in each plate. Cell viability was measured through three different cytotoxicity assays applied on the same set of cells according to a modified version [34,35] of a protocol described by Dayeh et al. [36]. The three cytotoxicity assays were the AlamarBlue assay, informing about alterations in the cellular metabolism; the CFDA-AM assay, which detects disruption of the plasma membrane integrity; and the Neutral Red assay which reflects lysosomal misfunctioning. The experimental results were compared to their corresponding control values using one-way repeated measures analysis of variance (ANOVA; *p* < 0.05) followed by a post hoc Dunnett’s test.

#### 2.4.8. Thermogravimetric Analysis (TGA)

The thermogram of the dried poplar CNF was recorded from room temperature to 600 °C by a steady rate of 10 °C min^−1^ under a nitrogen atmosphere in a Q-50 (TA Instrument Waters, New Castle, DE, USA) apparatus.

#### 2.4.9. Fourier-Transform Infrared Spectroscopy (FTIR)

The FTIR spectra of poplar pulp and dried CNF were performed by using a JASCO FT/IR 460 Plus spectrometer (Jasco Inc., Tokyo, Japan), equipped with an accessory single reflection diamond. The spectrum was obtained within the range of 600 to 4000 cm^−1^ wavenumber at a resolution of 1 cm^−1^ and 400 scans.

#### 2.4.10. Zeta Potential and Particle Size

Prior to these tests, a modification of poplar CNF was carried out by adding either 1M hydrochloric acid or 1M sodium hydroxide solutions in the case lower or higher pHs were targeted, respectively. Then, both zeta potential and particle size were measured using a Zetasizer Nano series (Nano ZS, Malvern, UK) with CNF concentrations of 0.05% wt. The measurements were repeated at least three times, showing mean values and standard deviations.

#### 2.4.11. Rheological Characterization

The rheological properties of the pH-modified poplar CNFs were measured by small-amplitude oscillatory shear (SAOS) and viscous flow tests. For that, frequencies from 0.03 to 100 rad·s^−1^ and increasing stepped shear rate ramps from 0.03 to 100 s^−1^ were respectively applied in both the Rheoscope (Thermo Haake, Vreden, Germany) and the ARES (Rheometric Scientific, Leaderhead, UK) rheometers. Rough plate–plate geometries of 20 and 25 mm diameter, respectively, and 1 mm gap were set as testing conditions. All rheological measurements were performed twice, showing mean values and standard deviations.

## 3. Results and Discussion

### 3.1. Poplar CNF Composition

In this work, cellulose nanofibers (CNFs) were produced from *P. alba* L., “PO-10-10-20”, an autochthonous genotype of the Mediterranean area. This material was planted following the Short Rotation Coppice (SRC) system. The chemical composition of the samples obtained at the third year of growth is shown in Table 1*. P. alba* L. “PO-10-10-20” raw material showed a similar glucan content than other materials from genus *Populus* previously studied [4,5,37]. Thus, Sannigrahi et al. [4] found a cellulose content of about 42–49% for different species and hybrids of poplar. In the same way, Ibarra et al. [5] referenced the glucan content in different poplar clones between 41.4 and 46.3%; and Martín-Davison et al. [37] between 35.5 and 40.4% for different poplar hybrids. With respect to hemicelluloses content, these authors have described a content of 16-23% [4,5,37]. Similar hemicelluloses content, 22.7%, was observed in the case of *P. alba* L. “PO-10-10-20”. The presence of around 25% of hemicelluloses has been reported to be beneficial for CNFs production, improving the efficiency of nanofibrillation [38]. The reason behind this finding is that hemicelluloses, mainly xylans, prevent microfibril aggregations by stabilizing the fiber suspension, which improves further fibrillation. Moreover, hemicelluloses contribute to the swelling of the fibers and this is one of the factors favoring the fibrillation process [20].

On the other hand, *P. alba* L. “PO-10-10-20” showed lower Klason lignin content (18.7%) than those previously shown for several hybrids and poplar clones (21–29%) [4,5,37]. In principle, this lower lignin content could also be beneficial for the use of this poplar material in the production of CNFs, because when the lignin content of the starting material is higher than 20%, the fibrillation yield decreases [39]. However, a residual amount of lignin is necessary to ensure good fibrillation as the antioxidant capacity of such lignin prevents the re-bonding of previously broken covalent bonds [40,41].

Kraft cooking and TCF bleaching processes were applied to obtain a material with the optimum quantities of lignin and hemicelluloses which favor subsequent fibrillation. After these processes, a decrease in lignin content (resulting in a 0.8% of Klason lignin), an increase in carbohydrates (reaching values of up to 74% in glucans and 24% in xylans), and a total elimination of extractives were observed in the resultant bleached pulp (Table 1). These values are very similar to those obtained by other authors using other kinds of biomass (including eucalypt as reference material) and who subsequently obtained CNFs with high yields [21,42,43,44].

Finally, as already demonstrated by other authors, during the TEMPO-ox reaction used as pre-treatment to obtain CNF, a delignification occurs (due to TEMPO itself and NaClO) together with a decrease of xylans [45,46]. This effect was observed herein for the resulting CNFs (Table 1), reaching lignin and xylans values of 0.4% and 16%, respectively. Consequently, the glucan content was risen up to 76.2%.

### 3.2. Poplar CNF Characterization

The degree of polymerization was measured for the pulp and CNF samples. A large decrease in this parameter (86.5%) was observed for the CNF sample, reaching a DPv value of 280 ± 81 vs. 2076 ± 51 found in the starting bleached pulp. Many authors have observed this strong decrease in the polymerization degree due to either physical or chemical pretreatments prior to microfluidization [18,47]. For example, for eucalypt, Fillat et al. [21] found a reduction of 87.53% in DPv using TEMPO-ox reaction as a pretreatment. However, Ibarra et al. [25] showed a reduction of 39% in DPv using physical pretreatment (PFI mill) prior to the microfluidization step. Normally, the decrease observed in this parameter is more pronounced in the case of TEMPO-ox reaction than in the physical pretreatments. This is because in the physical pretreatments case the reduction of DPv occurs due to shear forces generated during refining and microfluidization, while in the case of chemical treatment there is also cellulose degradation caused by TEMPO-ox reaction [21].

Regarding nanofibrillation yield, poplar CNF showed a value of 87.3% ± 8.1%, indicating that almost all of the fibers in the starting material have been transformed to nano-sized. The nanofibrillation yield values for CNFs obtained by chemical TEMPO-ox pretreatment tend to be higher (80–100%) than those obtained when only physical processes are used (10–60%) [21,25,44,47,48]. This is due to the fact that sodium carboxylate groups are formed during the TEMPO-ox reaction. Then, the negative charges generated on the fiber’s surface induce repulsive forces between them, favoring the subsequent microfluidization process [20]. Therefore, the carboxylate content of CNF samples is an important factor which exerts influence on the nanofibrillation yield [21]. In the case of poplar CNF, a carboxylate content of 1048 ± 128 µmol g^−1^ was found. This value is similar to those reported by other authors for CNF samples obtained from olive tree pruning (1038 µmol g^−1^), eucalypt (1043 µmol g^−1^) and elm (1178 µmol g^−1^), using also a TEMPO-ox pretreatment [21,44]. However, when only physical processes were used to produce CNFs, the carboxylate content values were lower than 100 µmol g^−1^ [44].

Nevertheless, the carboxylate content is not the only parameter that influences the nanofibrillation yield. Thus, among the authors who used TEMPO-ox pretreatment, Fillat et al. [21] showed values close to 96% in nanofibrillation yield for CNF obtained from eucalypt, while Jiménez-López et al. [44] obtained a nanofibrillation yield of 100% with the same raw material and pretreatment. Therefore, the difference in the nanofibrillation values found by these authors could be due to a higher number of passes in the microfluidizer [44]. On the other hand, Fillat et al. [21] found nanofibrillation yield values of 48% for CNF obtained from a bleached olive tree pruning pulp using the same process (including TEMPO-ox pretreatment and microfluidization). Therefore, not only the total applied process (including both pretreatment and the fibrillation process and their conditions) but also the starting material determines the nanofibrillation yield values of the resulting CNFs.

Optical transmittance is related to the light scattering of fibers with dimensions in the visible light range. Thus, a transmittance of 100% corresponds to highly fibrillated nanofibers [20,30]. From the wavelength scanning of the poplar CNF sample, lower transmittance was obtained at 400 nm compared with 700 nm. This effect could be due to the presence of bundles in the poplar CNF sample, which can absorb at 400 nm, but not at 700 nm [45]. Poplar CNF showed its highest transmittance at 700 nm (83%). This value is lower than that found by Fillat et al. [21] for eucalypt CNF (96%) and higher than that of olive tree pruning CNF (67%). It is known that the transmittance is directly related to the nanofibrillation yield; the more nano-size fibers are in a CNF sample, the higher is the transmittance. Accordingly, nanofibrillation yield values for poplar, eucalypt and olive tree pruning CNF samples were 87.3%, 97% and 48%, respectively [21].

**Figure 1:**
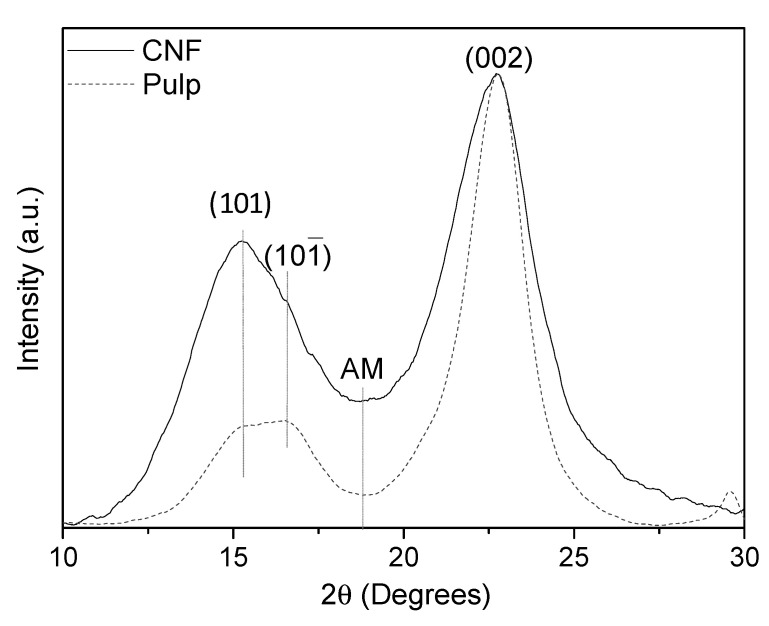
X-ray diffraction pattern of the poplar bleached pulp and CNF.

On the other hand, Figure 1 shows the X-ray diffraction patterns of the poplar pulp and CNF samples where the typical reflections corresponding to I_β_ cellulose allomorph can be observed. Thus, (101) and (002) reflections of the crystalline planes were observed at ~15.2 and ~22.7 2θ degrees and the (($10\bar{1}$)) reflection was indicated as a shoulder at ~16.5 2θ degrees. Moreover, the diffractograms did not show the three reflections at 12.2, 20.8 and 21.4θ degrees, corresponding to ($1\bar{1}0$), (110) and (200), respectively of cellulose II, suggesting that TEMPO-ox pretreatment followed by microfluidization did not induce the transformation of cellulose I into cellulose II. The crystallinity index and the crystallite size determined from these X-ray patterns were 93.8 ± 0.2% and 9.4 ± 0.4 nm for bleached pulp and 74.4 ± 1.6% and 7.2 ± 0.1 nm for CNF sample, respectively. In general, there is the notion that the TEMPO-ox pretreatment prior to microfluidization step can partially damage the crystalline cellulose structure [21], explaining the reduction of the crystallinity index from bleached pulp to CNF sample. Moreover, it is well known that the microfluidization step can affect the crystallinity of the resultant cellulose nanofibers due to the rupture of the highly ordered crystalline structure or to the hornification phenomenon of the cellulose nanofibers during this process [48,49]. The reduction of the crystallite size from bleached pulp to CNF during TEMPO-ox pretreatment and subsequent microfluidization has already been reported for bleached eucalypt and olive tree pruning pulps [21]. This effect is commonly attributed to the degradation of the amorphous phase during oxidation [50]. These crystallinity index and the crystallite size values described for poplar CNF are only slightly lower than those reported by Jiménez-López et al. [44] for TEMPO-ox CNF obtained from eucalypt (77 % and 8.2 nm) and elm (84 % and 9.4 nm). These values are also in the range of those reported by other authors for TEMPO-ox CNF obtained from different raw materials: 64–74% and 2.3–9.4 nm [17,20,44,51].

**Figure 2:**
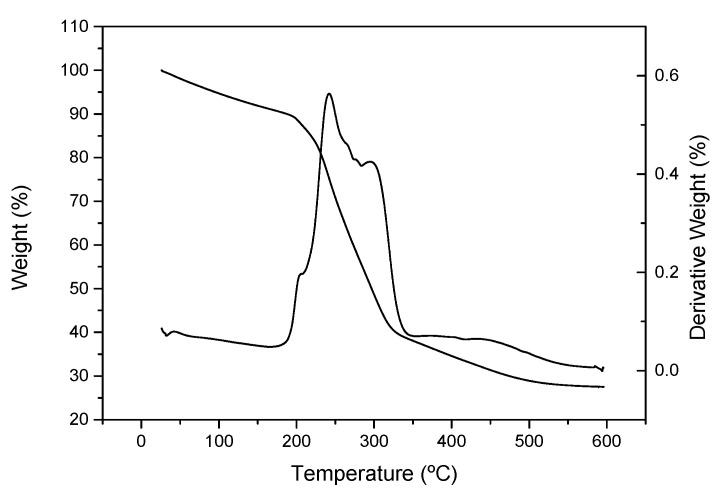
TGA of poplar CNF.

A thermogravimetric analysis (TGA) of the cellulose nanofibers from poplar was also performed (Figure 2). It can be observed that apart from the initial moisture loss, the poplar CNF sample exhibited outstanding thermal stability up to more than 200 °C, as previously reported by other authors with different CNFs [21,23]. The main weight losses take place around 250–300 °C with two incipient peaks centered at 242 and 295 °C. The first one is mainly due to dehydration of the cellulose chain and decarboxylation of sodium carboxylate groups, while the second one is mainly a consequence of the rupture of glycosidic linkages [52]. This last peak is shown at a lower temperature than that of cellulose pulp (typically between 300 °C and 350 °C), probably due to the lower diameter of nanofibers compared to cellulose fibers, which promote a faster degradation due to higher surface area exposed to heat [53,54]. From the different events, several characteristic parameters have been outlined, i.e., the temperature at which the degradation begins (T_onset_), the temperature at maximum derivative weight loss (T_max_) and the temperature at which the event is considered finished (T_final_). The weight loss during the events along with the remaining undegraded weight was also reported, all of them shown in Table 2. Similar T_onset_ and T_max_ were found for TEMPO-ox cellulose nanofibers from elm and eucalypt by Jiménez-López et al. [44]. However, these authors found higher T_final_. In agreement with other authors, the char residue obtained for TEMPO-ox poplar CNFs (28%) is higher than that typically found in non-chemical pretreated cellulose nanofibers (18–26%). This fact is due to the presence of carboxylate groups, which leads to the formation of carbon dioxide during decarboxylation that reacts with water (produced during dehydration of cellulose) forming sodium carbonate [44,55].

**Figure 3:**
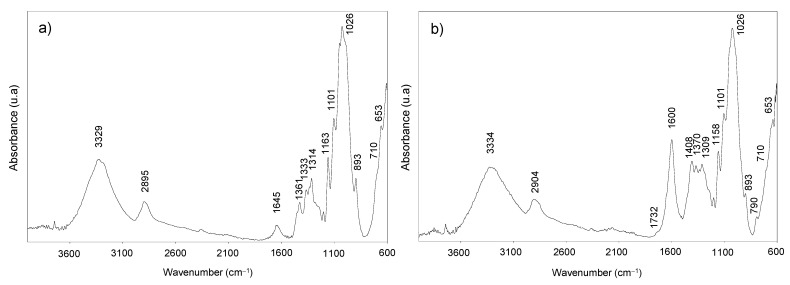
FTIR spectra of poplar pulp (**a**) and CNF (**b**).

Poplar pulp and dried CNF samples have also been analyzed by FTIR (Figure 3). The spectra show the typical bands that conform to the cellulose structure. The wide band centered around 3329 cm^−1^ is related to the hydroxyl groups, whereas the small band at 2895 cm^−1^ is due to asymmetric and symmetric C-H stretching vibrations [56]. The band detected at 1645 cm^−1^ in poplar pulp is attributed to the O-H stretching vibration of absorbed water. The three bands at 1163, 1101, and 1026 cm^−1^ observed in both CNF and pulp samples spectra can also be assigned to cellulose. Firstly, the band at 1163 cm^−1^ is related to pyranosyl rings among C-O asymmetric bands, the band at 1101 cm^−1^ is attributed to the C-OH skeletal vibration, while the band at 1026 cm^−1^ is related to C-O stretching vibration [57]. Moreover, the glycosidic bond vibration was observed at 893 cm^−1^ in both spectra, and the bands at 1361–1370 and 710 cm^−1^ also observed in both spectra indicate the presence of I_β_ cellulose allomorph [58], in agreement with X-ray diffraction analysis.

As the poplar CNF was obtained by the TEMPO-oxidation pretreatment, the stretching band from sodium carboxylate groups was prominently shown at 1600 cm^−1^ [21], supporting the oxidation of C6 primary hydroxyl groups of cellulose during the TEMPO-oxidation reaction. When these COONa groups are transformed into free carboxyl groups (COOH), this band would shift to 1732 cm^−1^, as it is observed in the CNF spectrum. Moreover, the band at 1408 cm^−1^ also indicates the presence of COONa, since it is associated with the C-O symmetric stretching of dissociated carboxyl groups [59].

### 3.3. Cytotoxicity Assays of Poplar CNF

**Figure 4:**
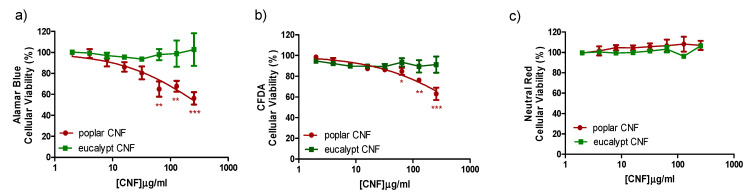
Effect of CNF samples on PLHC cell viability. Cells were exposed to increasing concentrations of CNFs for 72 h. Cytotoxicity of CNFs was assessed by means of the AlamarBlue assay (**a**), CFDA-AM assay (**b**), NRU assay (**c**). Points represent the mean and standard error of the mean (SEM) of at least three independent experiments. Statistically significant differences with respect to the vehicle control (one-way rmANOVA, Dunnett’s Post-hoc test) are indicated as followed: * *p* < 0.05, ** *p* < 0.01 and *** *p* < 0.001.

Although CNFs are isolated from cellulosic sources, biomasses with no or low toxicity, either the nanoscale or chemical modifications of nanofibers might influence their toxicity [60]. Then, the cytotoxicity assays of CNF samples obtained from *P. alba* L. “PO-10-10-20” and eucalypt (as reference CNF) were carried out. In general, the two CNF samples caused limited cytotoxicity to PLHC-1 cells so that an IC50 could not be calculated for any of the CNF samples with any of the cytotoxicity assays (Figure 4). In the case of the eucalypt CNF, no decrease of cell viability was detected with any of the tests so that even the calculation of an IC10 was not possible. Poplar CNF provoked a concentration-dependent decrease in cell viability allowing the calculation of IC10 values with AlamarBlue (IC10 = 10 µg mL^−1^) and CFDA-AM (IC10 = 60 µg mL^−1^) assays. No effect was observed at the lysosomal level by means of the Neutral Red assay. Such results suggest a very low toxicity of the tested CNF samples although additional in vivo experiments would be necessary to confirm the actual ecotoxicity of these substances. Of particular interest is the slight difference observed in the reduction of cell viability caused by both CNF samples (eucalypt and poplar). So far, considering the lack of significant differences observed in the properties of these cellulose nanofibers, it is difficult to hypothesize about the mechanisms underlying the slightly higher toxicity of poplar CNF. The investigation about the origin of this increase of cytotoxicity was out of the scope of the present study but it merits further efforts in the future.

Different studies have discussed the toxicity of different CNF samples, and in general none of them have shown evidence of the toxic effect on human health or the environment. Then, Vartiainen et al. [61] assayed the health and environmental safety of CNF isolated from birch via mechanical grinding. For that, mouse and human cells and luminescent bacteria were used as tested models, concluding that the tested CNF samples appeared hazardous to neither human health nor the ecological environment. Lopes et al. [62] assessed the in vitro biological responses elicited by wood-derived CNF samples (isolated from bleached sulfite softwood dissolving pulp by enzymatic hydrolysis combined with mechanical shearing and high-pressure homogenizationand), using human dermal fibroblasts, lung MRC-5 cells and THP-1 macrophage cells. Furthermore, they studied if the presence of surface-charged groups (i.e., carboxymethyl groups) on CNF samples could induce distinct biological responses. The assay data presented the absence of cytotoxic effects associated with the exposure to unmodified and carboxymethylated-modified CNFs. Finally, Alexandrescu et al. [63] produced nanofibrillated materials from *Eucalyptus* and *Pinus radiata* pulp fibers using a homogenizer without pretreatment and with TEMPO-ox pretreatment. Cytotoxicity tests were applied on the different CNF samples obtained, which showed that the nanofibrils do not exert acute toxic phenomena on the tested fibroblast cells (3T3 cells).

### 3.4. CNF Zeta Potential, Particle Size and Rheological Properties of Poplar CNF

**Figure 5:**
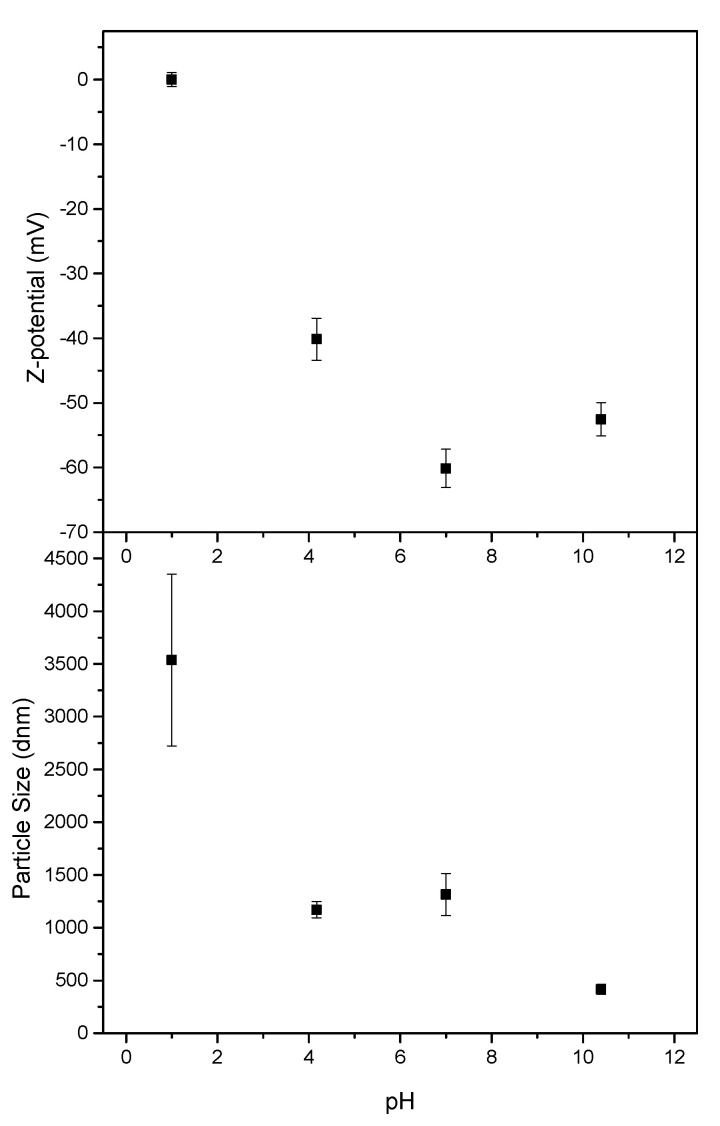
Zeta potential (**top**) and particle size (**bottom**) of nanocellulose dispersions as a function of pH.

Both zeta potential and particle size analysis of the different pH-modified poplar CNF samples were included in Figure 5. By slightly increasing or decreasing the pH from the neutral sample, higher values of zeta potential were obtained compared to the very low values obtained at neutral pH (−60 mV). Therefore, slightly poorer stability was exhibited, but still stable enough since the values were lower than −40 mV, which is the limit for colloidal stabilization [64]. Nonetheless, when the pH was reduced to 1, poplar CNFs gelled and formed clots even at the low 0.05 wt % solution concentration, exhibiting values of zeta potential close to 0 (isoelectric point), results which were in agreement with previous studies within the field [65,66].

However, the particle size did not follow the same trend. At the highest pH the particle size was significantly reduced, while only a small size decrease was observed at pH 4.17. It should be taken into account that the particle size determined here (by DLS assays) is not the real size of the nanofibers, but the hydrodynamic radius of an equivalent sphere that diffuses at the same rate as the nanofibers. Furthermore, this hydrodynamic radius will depend on the extent of particle-particle interactions in the aqueous suspension, which strongly depends on the particles’ concentration and their surface chemistry and resulting hydration shells [67]. Therefore, these particle sizes values give us only an estimation of the hydrodynamic size of the CNF samples but they do give us an indication of nanofibers aggregation caused by changes in pH. Thus, the particle size measured for the sample at pH 1 demonstrated the agglomeration seen with the naked eye, as a much larger size was reported. The general aggregation trend observed when reducing the pH is explained as a consequence of the cellulose carboxyl groups protonation, leading to a reduction in surface charge and hence diminishing electrostatic repulsions between fibers, also increasing the possibility of crosslinking through hydrogen bonding [49,66,68]. This is important to keep in mind if the application of the obtained poplar CNF requires significant pH changes.

**Figure 6:**
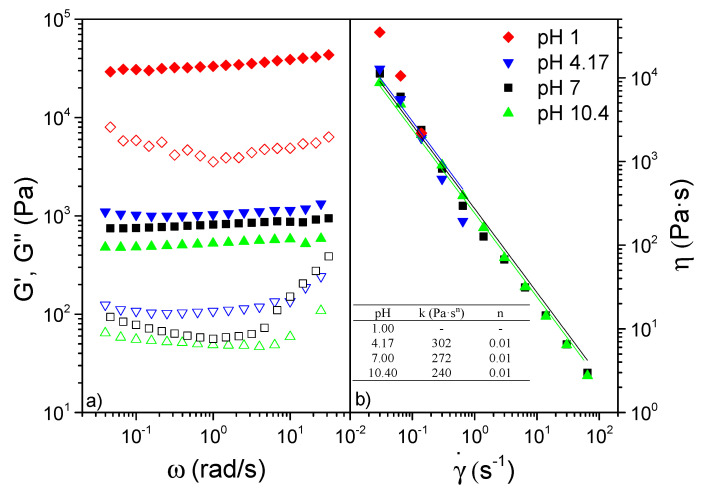
(**a**) Storage (filled symbols) and loss (empty symbols) functions and (**b**) viscosity of poplar CNF according to pH. Inset: values of K and n as a function of pH.

On the other hand, the rheological properties of the so-performed CNFs are also significantly influenced by the effect of pH, as can be clearly shown in Figure 6. Thus, the lower the pH, the higher the SAOS functions observed as previously reported Hujaya et al. [68] (Figure 6a). Nonetheless, this effect was not linear, as very close viscoelastic moduli values were obtained between pH 4.17, 7 and 10, unlike pH 1, which was able to raise viscoelastic functions in more than an order of magnitude (see Figure 6a). Regardless of the pH, the poplar CNFs presented a well-developed plateau zone, with predominant elastic characteristics due to the high separation of around an order of magnitude between storage (G’) and loss (G”) moduli, as already shown by other CNF-based hydrogels [68,69].

The subtle differences observed for viscoelastic functions between pHs 4.17, 7 and 10.4 were even lower when viscous flow measurements were considered (see Figure 6b). The shear-thinning behavior observed fitted properly the power-law model included in Equation (5):

(6) $$\eta =K\times {\dot{\gamma }}^{n-1}$$

where K and n are the consistency and flow indices, respectively. Once more, the stiffer samples generated when decreasing pH yielded higher values of the consistency index, whereas the flow index values close to 0 (see inset Figure 6b) demonstrate poplar CNFs to possess an extremely pronounced shear-thinning behavior [69,70]. Nonetheless, the extreme toughness exhibited by the pH 1 CNF did not allow a proper fit to be obtained as a consequence of the expulsion of the sample during the test.

## 4. Conclusions

This study has demonstrated the feasibility of obtaining cellulose nanofibers (CNFs) from fast-growing poplar, *P. alba* L., genotype “PO-10-10-20”, using chemical TEMPO-mediated oxidation followed by microfluidization. The resulting CNFs showed high carboxylate content, fibrillation yield, optical transmittance and crystallinity, properties very similar to those obtained with a reference raw material, eucalypt. Moreover, the rheological study of the poplar CNF at different pH values determined its potential in pH-sensitive hydrogel applications. Whereas pH from 4 to 10 did not produce significant changes in rheological behavior, a reduction of pH down to 1 led to an order-of-magnitude increase on the viscoelastic functions. Finally, a very low toxicity of the tested CNF samples was observed from in vitro assays.

## Figures and Tables

**Table 1:** Chemical composition of *P. alba* L. “PO-10-10-20” and its corresponding bleached pulp and CNF.

	Extractives (% o.d.p.)	Klason Lignin (% o.d.p.)	Soluble Lignin (% o.d.p.)	Glucans (% o.d.p.)	Xylans (% o.d.p.)
*P. alba* L. “PO-10-10-20”	6.3 ± 0.5	18.7 ± 0.1	4.0 ± 0.2	39.7 ± 0.4	22.7 ± 0.2
Bleached pulp	-	0.8 ± 0.6	0.4 ± 0.3	74.0 ± 2.1	24.0 ± 1.9
CNF	-	0.4 ± 0.3	0.2 ± 0.1	76.2 ± 1.7	16.0 ± 1.9

Mean values and standard deviations were calculated from triplicates.

**Table 2:** Thermogravimetric analysis results of poplar CNF.

	T_onset_ (°C)	T_max_ (°C)	T_final_ (°C)	∆W (%)	Residue (%)
Poplar CNF	218/270	242/295	256/320	32/40	28

## Data Availability

Not applicable.
